# Autophagy-related protein PlATG2 regulates the vegetative growth, sporangial cleavage, autophagosome formation, and pathogenicity of *peronophythora litchii*

**DOI:** 10.1080/21505594.2024.2322183

**Published:** 2024-03-04

**Authors:** Lin Lv, Chengdong Yang, Xue Zhang, Taixu Chen, Manfei Luo, Ge Yu, Qinghe Chen

**Affiliations:** a Hainan Yazhou Bay Seed Laboratory, College of Breeding and Multiplication (Sanya Institute of Breeding and Multiplication), Sanya, China; b Key Laboratory of Green Prevention and Control of Tropical Plant Diseases and Pests, Ministry of Education, School of Tropical Agriculture and Forestry, Hainan University, Haikou, China

**Keywords:** Autophagy, PlATG2, sporangial cleavage, autophagosome formation, pathogenicity, peronophythora litchii

## Abstract

Autophagy is an intracellular degradation process that is important for the development and pathogenicity of phytopathogenic fungi and for the defence response of plants. However, the molecular mechanisms underlying autophagy in the pathogenicity of the plant pathogenic oomycete *Peronophythora litchii*, the causal agent of litchi downy blight, have not been well characterized. In this study, the autophagy-related protein ATG2 homolog, PlATG2, was identified and characterized using a CRISPR/Cas9-mediated gene replacement strategy in *P. litchii*. A monodansylcadaverine (MDC) staining assay indicated that deletion of *PlATG2* abolished autophagosome formation. Infection assays demonstrated that Δ*Platg2* mutants showed significantly impaired pathogenicity in litchi leaves and fruits. Further studies have revealed that PlATG2 participates in radial growth and asexual/sexual development of *P. litchii*. Moreover, zoospore release and cytoplasmic cleavage of sporangia were considerably lower in the Δ*Platg2* mutants than in the wild-type strain by FM4–64 staining. Taken together, our results revealed that PlATG2 plays a pivotal role in vegetative growth, sporangia and oospore production, zoospore release, sporangial cleavage, and plant infection of *P. litchii*. This study advances our understanding of the pathogenicity mechanisms of the phytopathogenic oomycete *P. litchii* and is conducive to the development of effective control strategies.

## Introduction

Oomycetes form a diverse group of eukaryotic microorganisms that are superficially similar to filamentous fungi but are evolutionarily close to diatoms and brown algae in the stramenopiles [1,2]. Major oomycetes can cause devastating diseases in a wide range of economically important plants, resulting in enormous economic loss. For instance, *Phytophthora infestans*, a pathogen that causes the famous late blight of potato disease, *P. sojae* causes root rot in soybean, and sudden oak death resulting from *P. ramorum* [1,2]. In addition, litchi downy blight caused by *Peronophythora litchii* is the most destructive disease of litchi, resulting in enormous losses during production and postharvest [3–5]. However, the infection mechanisms of *P. litchii* are not well understood. Therefore, it is necessary to explore the mechanisms of pathogenicity to promote the development of more efficient means against litchi downy blight.

Autophagy is an intracellular process thought to occur in almost all eukaryotic microorganisms. Cytoplasmic contents are engulfed by double membrane-bound structures, called autophagosomes, and delivered into the lysosome/vacuole for degradation to promote cellular homoeostasis and survival during autophagy [6,7]. Autophagy is regulated by a group of autophagy-related genes (*ATGs*), and more than 40 *ATGs* have been identified in yeast, 19 of which are regarded as the core autophagy machinery involved in autophagosome formation [8–11]. ATG2 is one of the core autophagy machinery and a peripheral membrane-associated protein that is localized to the pre-autophagosomal structure (PAS). ATG2 can interact with phosphatidylinositol 3-phosphate (PtdIns(3)P)-binding protein ATG18 to form a complex to help ATG2 localize to PAS, whereas the interaction between ATG2 and ATG18 is independent of PtdIns(3)P [12–14]. In addition, ATG2 contains two membrane-binding domains that are important for the ATG2-ATG18 complex to tether PAS to the endoplasmic reticulum (ER) to initiate isolation membrane (IM) expansion during autophagosome formation [15]. However, IM formation is abrogated by a lack of the ATG2-ATG18 complex, although other ATG proteins are deposited at the PAS [14]. In *Saccharomyces cerevisiae*, ATG2 promotes autophagy and modulates ATG9 retrieval from PAS by interacting with ATG9 [16,17]. In mammals, ATG2A regulates ATG9A vesicle delivery and IM expansion by directly interacting with ATG9A at the mitochondria-associated endoplasmic reticulum (ER) membrane (MAM) during autophagosome biogenesis [18]. ATG2 specifically coordinates ATG9 and ATG18a trafficking to promote autophagosome closure in Arabidopsis [19–21]. These findings suggested that ATG2 plays an essential role in autophagosome biogenesis in different species.

Accumulating evidence indicates that autophagy plays an important role in vegetative growth, asexual/sexual reproduction, environmental stimuli, and pathogenicity in filamentous fungi and plants [22–27]. Kershaw and Talbot have demonstrated that deletion of any of the 16 genes, including *ATG2* associated with non-selective macroautophagy causes *Magnaporthe oryzae* to lose the ability to cause rice blast disease, due to defects in appressorium maturation [23]. In *Fusarium graminearum*, 28 *ATGs* deletion mutants, except the Δ*Fgatg17* mutant, showed differentially reduced radial growth, asexual/sexual development, pathogenicity, and deoxynivalenol (DON) production [25]. In the fungal pathogen *Cryptococcus neoformans*, the Δ*atg2* and Δ*atg6* strains displayed higher sensitivity to oxidative stress. Approximately half of the 22 Δ*atg* strains displayed significantly lower virulence in the *Galleria mellonella* model than in the wild type. Additionally, ATG1-ATG13, ATG11, ATG5-ATG12-ATG16, and ATG2-ATG18 complexes are required for autophagic flux due to cleavage of the green fluorescent protein (GFP) from ATG8 was hardly visualized in these mutants [28]. Taken together, these findings suggest that autophagy plays an indispensable role in vegetative growth, pathogenicity, and stress response, ATG2 positively regulates pathogenicity in pathogenic fungi.

Autophagy functions in the defence response of plants. In Arabidopsis, early senescence and excessive immunity-related programmed cell death (PCD) have been observed in *ATG2* and *ATG5*-defective strains. Deletion of *ATG2* also results in higher levels of reactive oxygen species (ROS) and an increased number of peroxisomes in guard cells [29,30]. Moreover, *ATG2* and *ATG18a*-defective mutants showed increased resistance to powdery mildew and dramatic mildew-induced cell death in Arabidopsis [31,32]. Hashimi et al. revealed that silencing of *GmATG2* accelerated the senescence phenotype, induced expression of pathogenesis-related gene 1 (*PR1*), and increased resistance to *Pseudomonas syringae pv. glycinea* (Psg) in soybean *Glycine max* [33]. Collectively, these findings suggest that autophagy plays an indispensable role in defence responses in plants. ATG2 negatively modulates the resistance of plants to pathogens.

In recent years, autophagy has been demonstrated to play a vital role in plant pathogenic oomycetes. Studies have shown that autophagy is essential for the development of *P. infestans* and *P. sojae* [34,35]. Deletion of *ATG6a* homologs *PsATG6a* and *PlATG6a* attenuated autophagosome formation and decreased the pathogenicity of *P. sojae* and *P. litchii* on soybean and litchi, respectively [34,36]. In addition, *PsATG2* displayed a higher transcription level during the infection stage in *P. sojae* [34], indicating that PsATG2 probably participates in the virulence of *P. sojae*. However, the functions of PlATG2 in vegetative growth, autophagosome formation, and pathogenicity of *P. litchii* are largely unclear. In this study, the ATG2 homolog PlATG2 was identified and knocked out using the CRISPR/Cas9-mediated gene replacement strategy. Phenotypic analysis revealed that PlATG2 plays an essential role in radial growth, asexual/sexual differentiation, sporangial cleavage, autophagosome formation, and infection in *P. litchii*.

## Materials and methods

### Phylogenetic analysis of ATG2 homologous proteins

Amino acid sequences of all ATG2 homologs were obtained from the website (https://fungidb.org/fungidb/app). The retrieved ATG2 protein sequences were submitted to an online website (http://pfam.xfam.org/search) to predict evolutionarily conserved functional domains. Multiple sequence alignments of ATG2 homologs from different species were performed with the ClustalW program, and a phylogenetic tree was constructed in MEGA 7.0, using the Maximum Likelihood method with 1000 bootstrap replicates.

#### P.Litchii *strain and growth conditions*

In this study, *P. litchii* strain SHS3 was used as the wild-type strain. The wild-type strain, a transformant that includes an empty vector (EV), was used as a control, *PlATG2* deletion mutant strains were routinely cultured on 10% (v/v) V8 agar plates at 25 °C in the dark. To determine the growth rate, all the indicated strains were cultivated on plates containing solid V8 medium in a 25 °C incubator in the dark. Individual mycelial disks, 5 mm in diameter, were cut from the edge of an actively growing colony and transferred to the centre of the V8 plates. The colony diameter was measured and photographed after 5 days. To understand asexual reproduction, various strains were grown on solid V8 plates for 5 days, and sporangia produced by these strains were washed with distilled water and counted using a haemocytometer under a microscope. Sporangia suspensions were sustained at 13°C for 0.5 and 2 h to promote zoospore release and to identify the zoospore release ability, respectively. For oospore formation, the tested strains were cultivated on V8 agar plates in a 25 °C incubator in darkness for 10 d. Three mycelial disks (diameter, 0.5 cm) around the inoculated mycelial plugs were selected, and five random fields for each disk at 10× magnification were used to calculate the number of oospores [37].

## Plasmid construction and target gene deletion

Two single guide RNA (sgRNAs) (Table S1) for targeting *PlATG2* gene were designed using the website EuPaGDT (http://grna.ctegd.uga.edu/) and annealed as previously described [38]. The two sgRNA oligonucleotides were then ligated to a linearized “all-in-one” plasmid (pYF515) by digestion with restriction endonucleases NheI and BsaI, which can express both the Cas9 gene and sgRNA cassettes simultaneously. The *eGFP* gene and ~ 1 kb upstream and downstream sequences of the *PlATG2* gene-coding region were amplified and constructed into the pBluescript II KS+ plasmid using the ClonExpress II One Step Cloning Kit (Vazyme), which was then used as donor DNA in the CRISPR/Cas9-mediated gene knockout strategy [38]. The primer pairs F1/OR (Table S1), located within the upstream of ~ 1 kb upstream of the *PlATG2* ORF and ORF, respectively, were selected to identify the deletion of *PlATG2* in the candidate transformants. The primer pair F1/eR (Table S1) was used to detect the gene replacement events. All primers used are listed in Table S1.

## Sensitivity to various stress

To understand the sensitivity of the ∆*Platg2* mutants to different stressors, the experimental strains were cultivated on the solid 10% V8 agar plates supplemented with or without 0.4 M KCl, 0.4 M Sorbitol, 2 mM H_2_O_2_, 0.004% SDS, or 400 μg/mL Congo Red (CR) in a 25°C incubator. The colony diameter was measured and photographed after 5 days. Growth inhibition rate (%) = (colony diameter on stress-free media − colony diameter on stress media)/colony diameter on stress-free media × 100%.

## Quantitative real time PCR

The wild type SHS3 and ∆*Platg2* mutants strains were cultivated in liquid V8 for 2 days, total RNA was extracted from mycelia. Reverse transcription PCR (RT-PCR) was then performed with RNA as template to obtain cDNA using the RT kit (Takara, RR047A). The expression levels were quantified using the TB Green kit (Takara, RR420A) and the primer pairs listed in Table S1. The *P. litchii* housekeeping gene *β-ACTIN* was used as endogenous reference gene. The data obtained from the former steps were finally calculated with 2^−*∆∆*CT^ method according to previous description [39]. The significant difference was calculated in the GraphPad Prism software with multiple t-tests from biological replicates (** = p ≤ 0.01).

## Microscopy

For cell biology assays, the Red-fluorescent FM4–64 dye (T13320, Invitrogen, USA) was used to visualize the cleavage system within the sporangia of *P. litchii* [40,41]. Briefly, sporangia produced by the tested strains were washed with distilled water and incubated at 13°C for 0.5 and 2 h, respectively, to promote the cytoplasmic cleavage of sporangia. The sporangia suspensions were stained with FM4–64 at a final concentration of 1 μg/mL for 5 min in the dark and then observed under a fluorescence microscope at different time points. Subsequently, the number of sporangia that did release zoospores was calculated using a fluorescence microscope. To observe nuclei distribution, sporangia suspensions were incubated at 13 °C for 15 min, stained using the blue fluorescent DAPI nucleic acid stain 4′, 6-diamidino-2-phenylindole (D3571, Invitrogen, USA) at a final concentration of 1 μg/mL for 5 min in the dark, and then visualized by fluorescence microscopy.

## Virulence assays

Virulence of all experimental strains was determined by mycelial plug inoculation of detached litchi leaves and fruits. The inoculated leaves and fruits were maintained at 80% humidity in black bags in a 25 °C incubator, and the typical symptomatic leaves and fruits were photographed 48 h post-inoculation (hpi). These experiments were independently replicated in triplicate. Statistical analyses were conducted using GraphPad Prism with multiple t-tests from three independent biological replicates at *p* ≤ 0.01.

## MDC staining

To determine autophagosome formation, the various strains were cultured in liquid 10% V8 medium for 48 h. Hyphae were washed three times with sterile distilled water and transferred into nitrogen starvation media containing 2 mM phenylmethylsulfonyl fluoride (PMSF) for 4 h. The hyphae were then stained with monodansylcadaverine (MDC) (D4008, Sigma) at a final concentration of 100 μM in phosphate-buffered saline (PBS) for 10 min in the dark, washed with PBS three times to remove excess dye, and viewed using fluorescence microscopy [34,42]. All the samples were kept on ice in the dark prior to microscopic observation.

## Results

### *Identification and deletion of ATG2 homolog in* P. litchii

**Figure 1.**
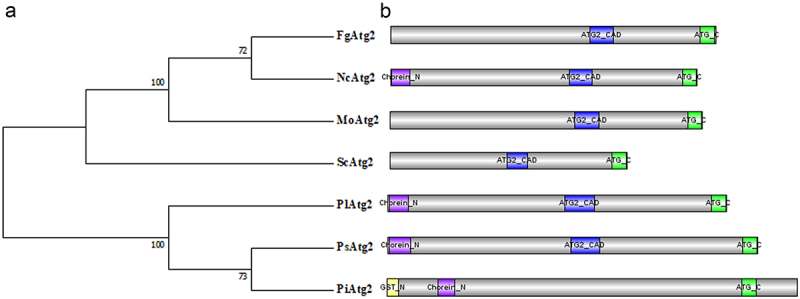
Identification of PlATG2 in *P. litchii.*

An ATG2 homolog was identified in *P. litchii* by a BLAST search using the amino acid sequence of yeast ATG2 as bait and designated PlATG2, which harbours 7728 base pairs without introns and encodes a protein with 2575 amino acids (aa). Functional domain analysis demonstrated that PlATG2 harbours a Chorein_N (N-terminal region of Chorein or VPS13) domain (12–135 aa), ATG2_CAD (Autophagy-related protein 2 CAD) motif (1187–1387 aa), and ATG_C (autophagy-related protein C terminal) domain (2169–2264 aa). Two or three of these domains in PlATG2 were also present in fungal and oomycete homologs of *S. cerevisiae*, *F. graminearum*, *M. oryzae*, *Neurospora crassa*, *P. sojae* and *P. infestans* (Figure 1b). Comparative phylogenetic analysis of ATG2 homologs revealed that PlATG2 is more closely related to its orthologs in *P. sojae* and *P. infestans* than in yeast and filamentous fungi (Figure 1a). These results indicated that ATG2 proteins may display similar and distinct biological functions in various species.

(a) Comparative phylogenetic analysis of ATG2 homologs from *S. cerevisiae*, *F. graminearum*, *M. oryzae*, *Neurospora crassa*, *P. sojae* and *P. infestans* was completed using MEGA7 software using the Maximum Likelihood method with 1000 bootstrap replications. (b) Conserved domains of ATG2 homologs in various species were predicted using a website (http://pfam.xfam.org/search).

To understand the role of PlATG2 in *P. litchii*, CRISPR/Cas9-mediated gene replacement technology [38] was used to generate *PlATG2* knockout mutants. The deletion mutants were verified by both PCR and sequencing, which displayed a 1.930 kb band with specific F1/eR primer pairs in the *PlATG2* knockout mutants and a 1.434 kb band with primer pairs F1/OR in the wild-type SHS3 (Supplementary Figure S1A-B). Sequencing further demonstrated that *PlATG2* was replaced by *eGFP* as donor DNA (Supplementary Figure S1C). Quantitative real time PCR (qrt-PCR) results showed that *PlATG2* is barely expressed in the *PlATG2* deletion mutants (Supplementary Figure S2). Transformants containing empty vectors (EV) were used as negative controls. Unfortunately, complementation strains of *PlATG2* deletion mutants have not been obtained by many attempts using the CRISPR/Cas9-mediated in situ complementation method [37].

## PlATG2 is critical for autophagy

**Figure 2.**
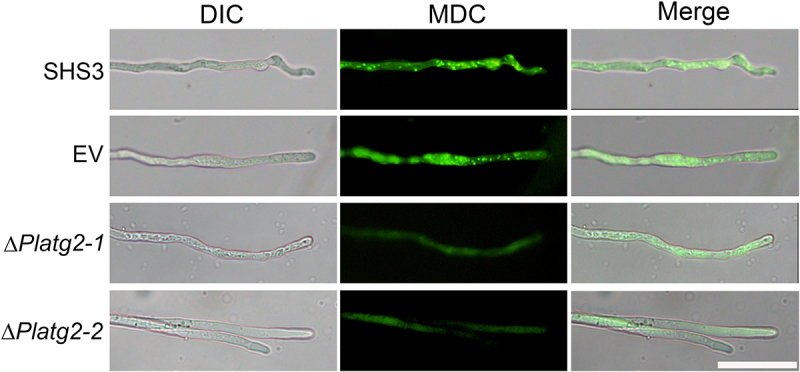
PlATG2 participates in autophagic pathway.

Deletion of *ATG6a* homologs *PsATG6a* and *PlATG6a* attenuated autophagosome formation and decreased the pathogenicity of *P. sojae* and *P. litchii* on soybean and litchi, respectively [34,36]. To gain insight into the function of PlATG2 in autophagy, the experimental strains were grown in 10% V8 medium and transferred to nitrogen-starvation conditions for 4 h. Hyphae were then stained with monodansylcadaverine (MDC) to indicate autophagosome formation. As shown in Figure 2, Δ*Platg2* mutants exhibited minimal MDC fluorescence in the hyphae. In contrast, MDC-stained autophagosomes were clearly present in the hyphae of wild-type and EV strains (Figure 2), indicating that PlATG2 plays a critical role in the autophagic pathway.

Deletion of *PlATG2* blocks autophagosome formation. The various strains were grown in liquid 10%V8 medium for 48 h and transferred to nitrogen starvation conditions with 2 mM phenylmethylsulfonyl fluoride (PMSF) for 4 h. Hyphae were stained with 100 μM MDC in the dark for 10 min and washed with distilled water three times. Images were captured using a fluorescence microscope. Bar = 20 μm.

## PlATG2 is involved in vegetative growth and tolerance to stressors

**Figure 3.**
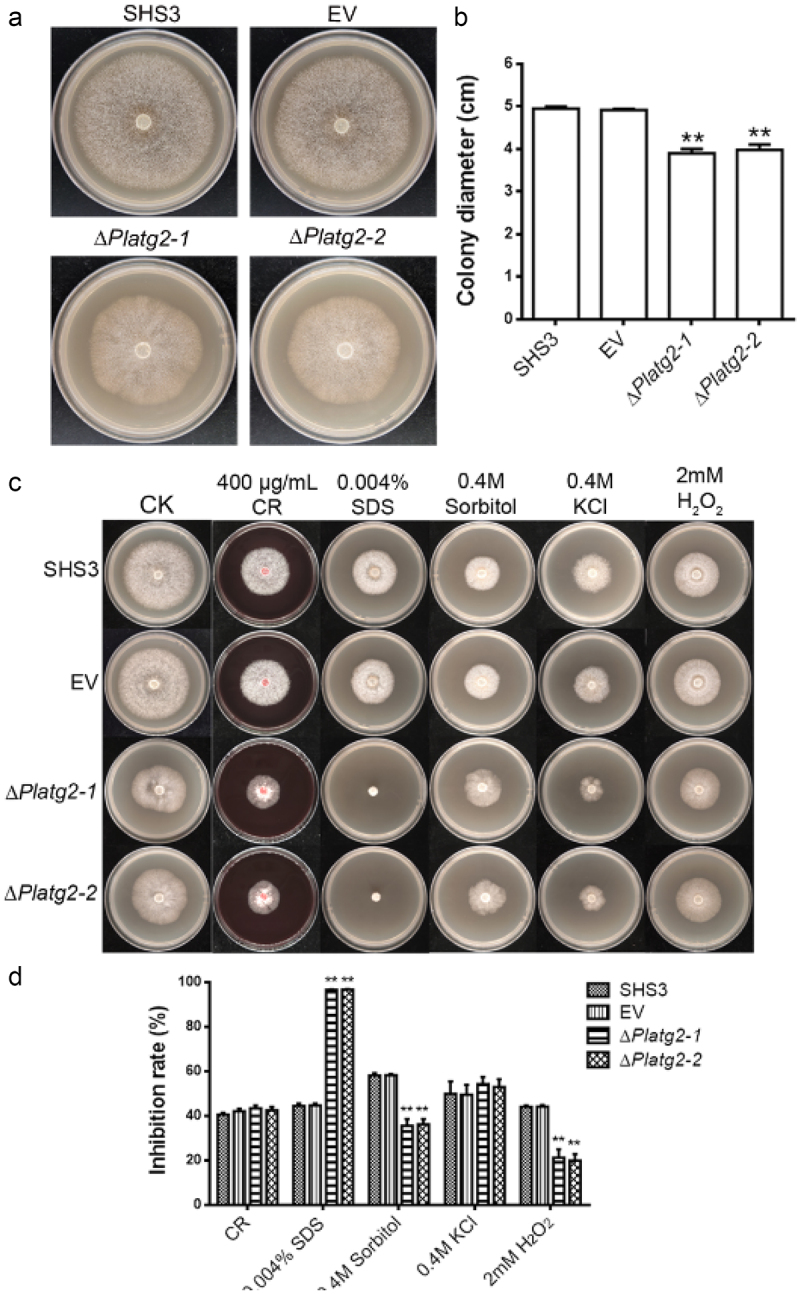
Deletion of *PlATG2* decrease the radial growth of *P. litchii.*

To characterize the role of PlATG2 in the vegetative growth of *P. litchii*, wild-type SHS3, EV, and *PlATG2* deletion mutant strains were cultured on solid 10% V8 agar plates for 5 days, and colony diameters were then analyzed. The results showed that the mycelial growth of the Δ*Platg2* mutants was reduced compared to that of the wild-type and EV strains (Figure 2a). The colony diameter of the Δ*Platg2* mutant was approximately 4.0 cm, which was decreased by 20% compared to 5.0 cm of the wild-type strain (Figure 3b). Furthermore, the Δ*Platg2* mutants showed fewer aerial hyphae than SHS3 (Figure 3a), suggesting that PlATG2 plays an important role in the vegetative growth of *P. litchii*.

(a) Wild-type SHS3, EV, and *PlATG2* deletion mutant strains were grown on 10%V8 agar plates in a 25 °C incubator and photographed after 5 days inoculation. (b) Colony diameters of the examined strains were measured after 5 days of inoculation. Significant differences between the ∆*Platg2* mutants and the wild-type strain were analysed using multiple t-tests in GraphPad Prism with three biological repeats at *p* ≤ 0.01. (c) Wild-type SHS3, EV, and *PlATG2* deletion mutant strains were grown on 10%V8 agar plates with various stressors in a 25 °C incubator and photographed after 5 days inoculation. (d) The inhibition rate was calculated according to the following formula: (colony diameter without stressors − colony diameter with stressors)/colony diameter without stressors. Significant differences between the ∆*Platg2* mutants and the wild-type strain were analysed using multiple t-tests in GraphPad Prism with three biological repeats at *p* ≤ 0.01.

Next, we tested the sensitivity of the examined strains to various stressors. Wild-type, EV, and *PlATG2* deletion strains were cultivated on solid 10% V8 agar plates supplemented with or without 0.4 M KCl, 0.4 M Sorbitol, 2 mM H_2_O_2_, 0.004% SDS, or 400 μg/mL Congo Red (CR) in a 25 °C incubator. The colony diameter was measured after 5 days. We found that Δ*Platg2* mutants were highly sensitive to SDS (cell wall stress) compared to the wild-type and EV strains. Surprisingly, deletion of *PlATG2* resulted in high tolerance to sorbitol (osmotic stress) and H_2_O_2_ (oxidative stress). The sensitivity of Δ*Platg2* mutants to KCl (salt stress) and CR (cell wall stress) was comparable to that of the wild-type and EV strains (Figure 3c,d). These results implied that PlATG2 plays a distinct role in the response to various stresses.

## PlATG2 is important for asexual and sexual development

**Figure 4.**
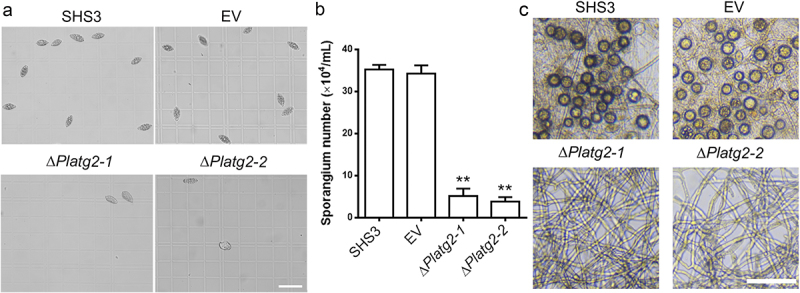
PlATG2 is involved in asexual and sexual reproduction.

Sporangium produced by *P. litchii* plays an important role in infection of litchi [43,44]. Next, we examined sporangium production in Δ*Platg2* mutants. After 5 days of growth on solid 10% V8 medium, the sporangia of the tested strains were collected and counted. Our results showed that sporangia of the Δ*Platg2* mutants were significantly reduced compared to the SHS3 and EV strains (Figure 4a). Average 4.5 × 10^4^ ml^−1^ sporangia were produced by the *PlATG2* deletion mutants in comparison to 3.5 × 10^5^ ml^−1^ and 3.4 × 10^5^ ml^−1^ sporangia formed by SHS3 and EV strains, respectively (Figure 4b). In addition to sporangia, the oospores of oomycetes are believed to be an inoculum in the disease cycle [43,44]. Thus, we investigated the sexual development of the various strains on 10% V8 agar plates and found that oospore production was completely abolished in the Δ*Platg2* mutants, while a large number of oospores were formed in the wild-type and EV strains (Figure 4c). Collectively, these results showed that PlATG2 is important for normal radial growth and asexual and sexual development in *P. litchii*.

(a-b) Sporangia produced by the wild-type SHS3, EV, and the *PlATG2* deletion mutant strains were captured and counted under a light microscope. Bar = 200 μm. Significant differences between the ∆*Platg2* mutants and the wild-type strain were analysed by multiple t-tests in a GraphPad prism with three biological repeats at *p* ≤ 0.01. (c) Oospores formed by the wild-type SHS3, EV, and the *PlATG2* deletion mutant strains were visualized under a light microscope after 10 days inoculation. Bar = 200 μm.

## PlATG2 is required for zoosporogenesis and sporangia cleavage during zoospore development

**Figure 5.**
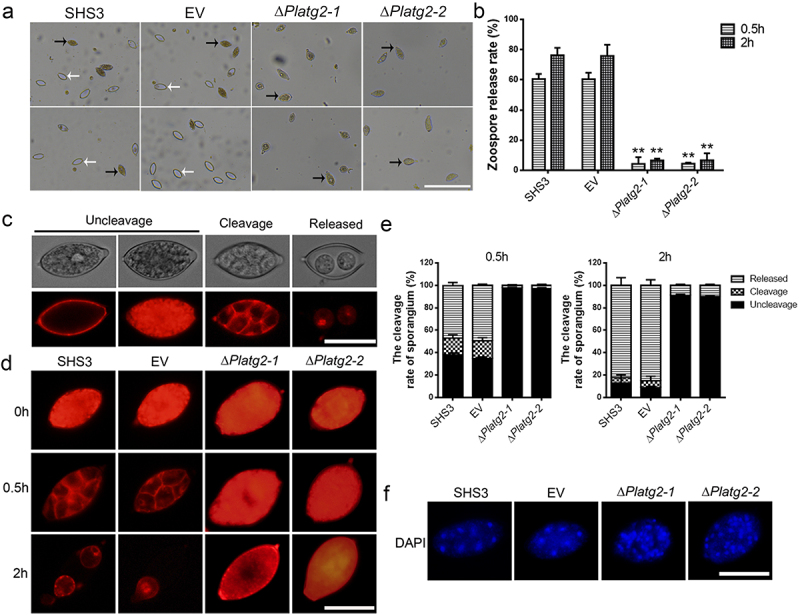
Deletion of *PlATG2* disrupted zoospore production and sporangia cleavage during zoospore development.

Sporangia produced by oomycetes can release zoospores, which are considered important factors in the initial infection and spreading of the pathogen [45]. To investigate zoospore production, sporangia produced by the wild-type SHS3, EV, and *PlATG2* deletion mutant strains were cultured at low temperature for 0.5 and 2 h, respectively. The results demonstrated that the deletion of *PlATG2* drastically decreased zoospore release compared to the wild-type and EV strains (Figure 5a). Approximately 60% of sporangia from the wild-type and EV strains released zoospores at 0.5 h. However, less than 7% of sporangia produced by the Δ*Platg2* mutants released zoospores even after 2 h (Figure 5b).

(a) Sporangia produced by the tested strains were incubated at low temperature (13°C) for 0.5 and 2 h to promote zoospore release. The pictures were captured under a light microscope. Bar = 200 μm. White arrows indicate the sporangia that has released zoospores, while black arrows indicate sporangia that have not. (b) Zoospore release rate of the indicated strains were counted after 0.5 and 2 h induction under low temperature. Significant differences between the ∆*Platg2* mutants and the wild-type strain were calculated by multiple t-tests in a GraphPad prism with three biological repeats at *p* ≤ 0.01. (c) Three types of sporangia (uncleavage, cleavage, and released) were observed by FM4-64 staining after low temperature induction under fluorescence microscopy. Bar = 20 μm. (d) Sporangial cleavage of the experimental strains were observed by FM4-64 staining at indicated time points using a fluorescence microscopy. Bar = 20 μm. (e) The rate of sporangial cleavage in the tested strains were calculated under a fluorescence microscopy. Significant differences between the ∆*Platg2* mutants and the wild-type strain were calculated by multiple t-tests in a GraphPad prism with three biological repeats at *p* ≤ 0.01. Bar = 20 μm. (f) The nuclei distributions in sporangium of the wild-type, EV, and the ∆*Platg2* mutants were visualized with DAPI staining by a fluorescence microscopy. Bar = 20 μm.

Zoosporogenesis requires the cleavage of the sporangial cytoplasm by membrane networks in oomycetes [46]. Therefore, sporangial cleavage in the indicated strains was observed using FM4–64 and DAPI staining. Our results indicated that the deletion of *PlATG2* impaired the cleavage of the sporangial cytoplasm. As shown in Figure 5c-e, FM4–64 was mostly on the differentiated cytoplasm of sporangia (cleavage) and released zoospores (released) in the wild-type and EV strains under low temperature for 0.5 and 2 h, respectively. However, FM4–64 was mainly present in the undifferentiated cytoplasm of sporangia (uncleavage) in the Δ*Platg2* mutants at low temperatures, even after 2 h (Figure 5c-e). DAPI staining further revealed that the nuclei in the sporangia of the wild-type and EV strains were regularly spaced, and the cytoplasm differentiated to promote zoospore formation. Conversely, the sporangial cytoplasm in the Δ*Platg2* mutants remained undifferentiated and the nuclei were disordered (Figure 5f). These findings collectively revealed that PlATG2 is essential for sporangia cleavage during zoosporogenesis and that deletion of *PlATG2* contributed to defects in zoospore release in *P. litchii*.

## PlATG2 is indispensable for pathogenicity of *P. litchii*

**Figure 6.**
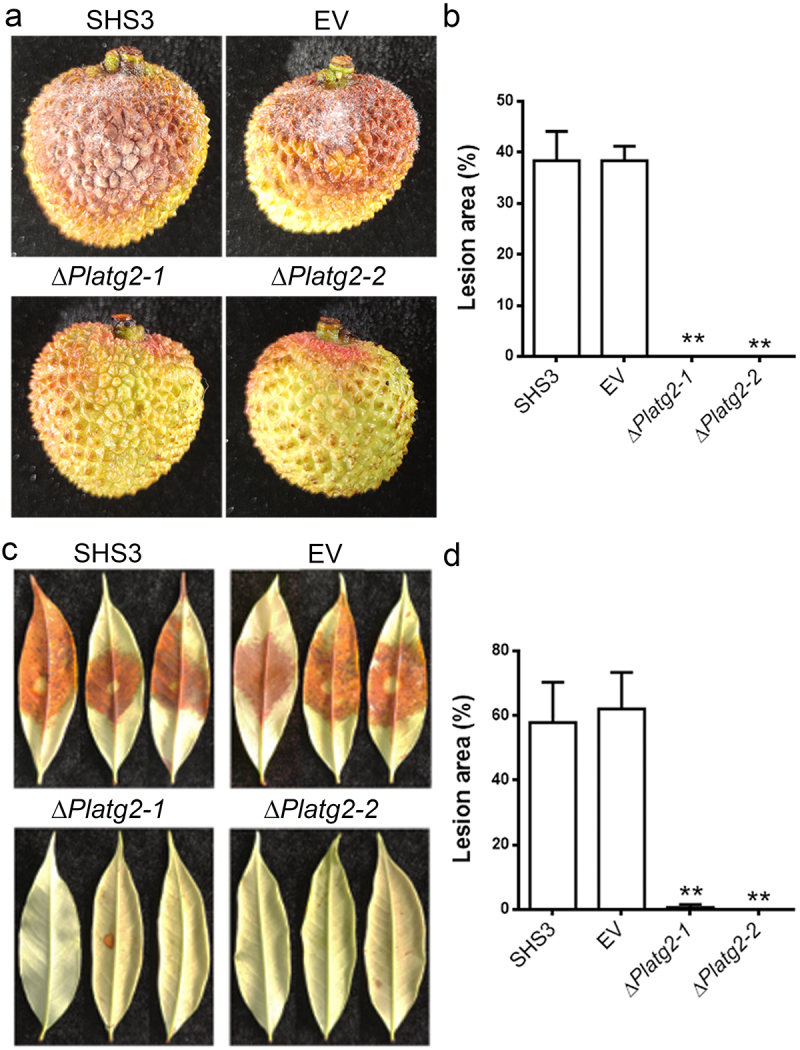
Deletion of *PlATG2* abolished pathogenicity of *P. litchii.*

To elucidate whether PlATG2 plays an important role in the pathogenicity of *P. litchii*, mycelial plugs of the wild-type SHS3, EV, and *PlATG2* deletion mutants were inoculated on litchi fruits under high humidity for 3 days. The results showed that no lesions were formed on the fruits inoculated with the Δ*Platg2* mutants, whereas approximately 40% of the area on the fruits produced typical symptoms by the wild-type and EV strain inoculation (Figure 6a,b). Moreover, pathogenicity assays were also conducted on detached tender litchi leaves, and the results were similar to those of fruits (Figure 6c,d). These results indicated that PlATG2 is indispensable for the pathogenicity of *P. litchii*.

(a) The pathogenicity of ∆*Platg2* mutants in litchi fruits was blocked. Mycelial plugs of the wild-type, EV, and ∆*Platg2* strains were inoculated on the litchi fruits. Images were taken 3 days post-inoculation (dpi). (b) The lesion area on the fruits inoculated with the examined strains was counted after 3 dpi. Significant differences between different strains were calculated using multiple t-tests in a GraphPad prism with three biological repeats at *p* ≤ 0.01. (C) Pathogenicity of ∆*Platg2* mutants in tender litchi leaves was disrupted. Mycelial plugs of the corresponding strains were inoculated on tender litchi leaves. Photographs were taken and the lesion area was measured after 3 dpi. Significant differences in various strains were calculated using multiple t-tests in GraphPad Prism with three independent replicates at *p* ≤ 0.01.

## Discussion

In this study, an ATG2 homolog from *S. cerevisiae* was identified in *P. litchii* using BLASTN. Using the CRISPR/Cas9-mediated gene replacement technique, the predicted *P. litchii PlATG2* gene was functionally analysed at a relative global scale. We found that PlATG2 was essential for autophagosome formation in *P. litchii*. In addition, we found that PlATG2 is also important for radial growth, asexual/sexual reproduction, sporangial cleavage, and virulence in *P. litchii*. Overall, our findings reveal that PlATG2 plays a critical role in the pleiotropic functions of *P. litchii*.

It has been shown that ATG2 is a autophagy-related protein which participates in autophagosome formation and Cvt pathway. In yeast, disruption of ATG2 blocks the autophagy/Cvt pathway. An N-terminal mutant of ATG2 (G83E) partially diminished the autophagic process compared to the null and wild-type cells, suggesting that the N-terminus of ScATG2 is important for autophagosome formation [47,48]. In mammals, simultaneous silencing of ATG2A and ATG2B (ATG2 homologues) compromises autophagosome formation and increases the number and size of lipid droplets [49]. The dysfunction of ATG2 suppressed autophagy initiation or autophagosome formation, resulting in autophagy-deficient phenotypes in Arabidopsis [30,31]. Recently, Hashimi et al. found that silencing GmATG2 contributed to the accumulation of ATG8 protein and polyubiquitinated proteins in soybean plants, indicating that autophagic degradation is disrupted in GmATG2-silenced soybean [33]. In line with previous results, our findings indicated that the deletion of *PlATG2* also abolished autophagosome formation by MDC staining in *P. litchii*. Collectively, these data illustrate that the function of ATG2 in autophagosome formation is conserved among different species. However, the underlying molecular mechanisms of PlATG2 in the autophagic pathway need to be further elucidated by identifying the interacting proteins in future studies.

Autophagy is an evolutionarily conserved process in all eukaryotes, from yeast to humans, and a major and non-selective degradation pathway for proteins and cytoplasmic organelles responsible for maintaining intracellular homoeostasis in response to environmental stimuli [50–52]. Accumulating evidence has revealed that autophagy is necessary for mycelial growth, environmental stress, asexual/sexual reproduction, and virulence in plant pathogenic fungi [22,23,25,53–55]. Deletion of autophagy-related genes differentially influences vegetative growth in filamentous fungi. Disruption of *MgATG2* results in sparse aerial hyphae compared to the wild-type strain Guy11 in *Magnaporthe grisea* [56]. In *F. graminearum*, the vegetative growth of the 18 *ATGs* deletion mutants was significantly lower than that of the wild-type strain. The development of aerial mycelia in the Δ*Fgatg2* mutant was significantly attenuated. However, the growth rate of *FgATG2* deletion mutant is not significantly different from that of the wild-type strain [25]. In the fungal pathogen *Cryptococcus neoformans*, ATG6 and ATG14–03 markedly compromised the growth of *C. neoformans* at 37 °C, whereas *ATG2*-defective strain had no adverse influence on the growth of *C. neoformans* [28]. In recent years, several lines of evidence have indicated that autophagy plays an essential role in radial growth of oomycetes [34,36]. In our study, PlATG2, an ATG2 homolog, was involved in the vegetative growth of *P. litchii*. The Δ*Platg2* mutants showed reduced mycelial growth (reduced by 20%) and sparse aerial hyphae on 10%V8 agar medium in comparison to the wild-type strain. Taken together, these findings demonstrate that the autophagy-related protein ATG2 plays similar and different roles in mycelial growth in various species.

Numerous studies have revealed that autophagy plays an essential role in the virulence of plant-pathogenic fungi. In *M. oryzae*, deletion of 16 autophagy-related genes (*ATGs*), including *MoATG2* important for non-selective autophagy, dramatically or completely blocks the infection of pathogens on plants, due to functional defects of the appressorium that fail to penetrate the plant cuticle [23]. 28 *ATGs* include *FgATG2*, except *FgATG17*, play pivotal roles in DON production and pathogenicity of *F. graminearum*. Loss of these genes differentially prevents the pathogen from causing Fusarium head blight in wheat due to the reduced DON production and virulence in *F. graminearum* [25]. In addition, the loss of *CnATG2* significantly suppressed the virulence of *C. neoformans* in the *Galleria mellonella* model compared to that in the wild type [28]. In the present study, *PlATG2* deletion mutants also displayed defects in autophagosome formation and pathogenicity in *P. litchii*. Diseased symptoms were hardly observed on the litchi leaves and fruits inoculated with the Δ*Fgatg2* mutants, whereas typical symptoms were clearly visualized on the leaves and fruits inoculated with the wild-type strain. These data collectively demonstrated that, similar to ATG2 homologs from various fungus, PlATG2 involved autophagy plays an indispensable role in the infection of *P. litchii*. Conversely, autophagy has been reported to play a negative role in plant pathogen resistance. The *atg18a* and *atg2* mutants show early senescence phenotypes, increased resistance to powdery mildew, and tremendous mildew-induced cell death in Arabidopsis [31,32]. In agreement with this, silencing of *GmATG2* resulted in significantly increased resistance to *Pseudomonas syringae pv. glycinea (Psg)* in soybean [33]. These findings illustrate that the autophagy-related protein ATG2 negatively regulates plant resistance to pathogens. Recent studies have revealed that effectors secreted by the pathogens can target the autophagy-related proteins to enhance pathogens progression in plants [57–59]. Taken together, these findings provide insights into the versatile role of autophagy-related proteins in different species and are conducive to further exploring the function of autophagy in the pathogenicity of oomycetes.

Sporangia, zoospores, and oospores are important structures for the diffusion and infection of oomycetes [43,60]. In *P. sojae*, deletion of *PsATG6a* compromises autophagosome formation, sporangia production, zoospore release, and infection of the host [34]. Wang et al. showed that PlATG6a is involved in autophagosome formation, sporangia and oospore production, zoospore release, and virulence in litchi [36]. These results indicate that autophagy plays a critical role in the asexual and sexual development of oomycetes. In our study, *PlATG2*-deficient mutants showed reduced sporangia production and loss of oospore production compared with the wild-type strain. We further found that the loss of *PlATG2* remarkably undermines sporangial cleavage to inhibit zoospore release. Defects in asexual/sexual reproduction in Δ*Platg2* mutants may be one of the reasons for the decreased pathogenicity of *P. litchii*. Therefore, we infer that PlATG2 also possibly modulates asexual/sexual development by autophagy to enhance the infection of *P. litchii*. It is necessary to further explore how PlATG2 regulates asexual/sexual reproduction via the autophagy pathway to influence the virulence of *P. litchii*.

In summary, we have identified and investigated PlATG2, an autophagy-related protein that is required for autophagosome formation and is thus responsible for vegetative growth, asexual/sexual development, and pathogenicity of *P. litchii*. Future studies should focus on the identification of more autophagy-related proteins and the molecular mechanisms by which these proteins modulate the pathogenicity of *P. litchii*.

## Supplementary Material

PlAtg2 supplementary materialclean.docx

## Data Availability

All datasets presented in this study are included in the article/SupplementaryMaterial.

## References

[1] Kamoun S. Molecular genetics of pathogenic oomycetes. Eukaryot Cell. 2003;2(2):191–13. doi: 10.1128/EC.2.2.191-199.2003 12684368 PMC154851

[2] Kamoun S, Furzer O, Jones JD, et al. The top 10 oomycete pathogens in molecular plant pathology. Molecular Plant Pathology. 2015;16(4):413–434. doi: 10.1111/mpp.12190 25178392 PMC6638381

[3] Kao CW, Leu LS. Sporangium germination of *peronophythora litchii*, the causal organism of litchi downy blight. Mycologia. 1980;72(4):737–748. doi: 10.1080/00275514.1980.12021242

[4] Wang HC, Sun HY, Stammler G, et al. Baseline and differential sensitivity of *peronophythora litchii* (lychee downy blight) to three carboxylic acid amide fungicides. Plant Pathology. 2009;58(3):571–576. doi: 10.1111/j.1365-3059.2008.01990.x

[5] Xu L, Xue J, Wu P, et al. Antifungal activity of hypothemycin against *peronophythora litchii* in vitro and in vivo. J Agric Food Chem. 2013;61(42):10091–10095. doi: 10.1021/jf4030882 24106914

[6] Carlsson SR, Simonsen A. Membrane dynamics in autophagosome biogenesis. J Cell Sci. 2015;128:193–205. doi: 10.1242/jcs.141036 25568151

[7] Huang WP, Klionsky DJ. Autophagy in yeast: a review of the molecular machinery. Cell Struct Funct. 2002;27(6):409–420. doi: 10.1247/csf.27.409 12576634

[8] Fukuda T, Furukawa K, Maruyama T, et al. Mitofissin: a novel mitochondrial fission protein that facilitates mitophagy. Autophagy. 2023a;19(11):1–3. doi: 10.1080/15548627.2023.2237343 37455477 PMC10549205

[9] Fukuda T, Furukawa K, Maruyama T, et al. The mitochondrial intermembrane space protein mitofissin drives mitochondrial fission required for mitophagy. Mol Cell. 2023b;83(12):2045–2058 e2049. doi: 10.1016/j.molcel.2023.04.022 37192628 PMC10330776

[10] Fukuda T, Kanki T. Atg43, a novel autophagy-related protein, serves as a mitophagy receptor to bridge mitochondria with phagophores in fission yeast. Autophagy. 2021;17(3):826–827. doi: 10.1080/15548627.2021.1874662 33475472 PMC8032225

[11] Suzuki K, Ohsumi Y. Current knowledge of the pre-autophagosomal structure (PAS). FEBS Lett. 2010;584(7):1280–1286. doi: 10.1016/j.febslet.2010.02.001 20138172

[12] Jiang Z, Zhu L, Wang Q, et al. Autophagy-related 2 regulates chlorophyll degradation under abiotic stress conditions in Arabidopsis. Int J Mol Sci. 2020;21(12):4515. doi: 10.3390/ijms21124515 32630439 PMC7350272

[13] Obara K, Sekito T, Niimi K, et al. The Atg18-Atg2 complex is recruited to autophagic membranes via phosphatidylinositol 3-phosphate and exerts an essential function. J Biol Chem. 2008;283(35):23972–23980. doi: 10.1074/jbc.M803180200 18586673 PMC3259791

[14] Suzuki K, Kubota Y, Sekito T, et al. Hierarchy of Atg proteins in pre-autophagosomal structure organization. Genes Cells. 2007;12(2):209–218. doi: 10.1111/j.1365-2443.2007.01050.x 17295840

[15] Kotani T, Kirisako H, Koizumi M, et al. The Atg2-Atg18 complex tethers pre-autophagosomal membranes to the endoplasmic reticulum for autophagosome formation. Proc Natl Acad Sci USA. 2018;115(41):10363–10368. doi: 10.1073/pnas.1806727115 30254161 PMC6187169

[16] Feng Y, He D, Yao Z, et al. The machinery of macroautophagy. Cell Res. 2014;24(1):24–41. doi: 10.1038/cr.2013.168 24366339 PMC3879710

[17] Gomez-Sanchez R, Rose J, Guimaraes R, et al. Atg9 establishes Atg2-dependent contact sites between the endoplasmic reticulum and phagophores. J Cell Bio. 2018;217(8):2743–2763. doi: 10.1083/jcb.201710116 29848619 PMC6080931

[18] Tang Z, Takahashi Y, He H, et al. TOM40 targets Atg2 to mitochondria-associated ER membranes for phagophore expansion. Cell Rep. 2019;28(7):1744–1757 e1745. doi: 10.1016/j.celrep.2019.07.036 31412244 PMC6701867

[19] Kang S, Shin KD, Kim JH, et al. Autophagy-related (ATG) 11, ATG9 and the phosphatidylinositol 3-kinase control ATG2-mediated formation of autophagosomes in arabidopsis. Plant Cell Rep. 2018;37(4):653–664. doi: 10.1007/s00299-018-2258-9 29350244

[20] Luo M, Law KC, He Y, et al. Arabidopsis autophagy-related 2 is essential for ATG18a and ATG9 trafficking during autophagosome closure. Plant Physiol kiad. 2023;287(1):304–321. doi: 10.1093/plphys/kiad287 37195145

[21] Mishra D. Closing the loop: three musketeers of autophagy-ATG2, ATG18a, and ATG9. Plant Physiol kiad. 2023;369(1):177–178. doi: 10.1093/plphys/kiad369 PMC10469353 37379563

[22] Asakura M, Ninomiya S, Sugimoto M, et al. Atg26-mediated pexophagy is required for host invasion by the plant pathogenic fungus *Colletotrichum orbiculare*. Plant Cell. 2009;21(4):1291–1304. doi: 10.1105/tpc.108.060996 19363139 PMC2685618

[23] Kershaw MJ, Talbot NJ. Genome-wide functional analysis reveals that infection-associated fungal autophagy is necessary for rice blast disease. Proc Natl Acad Sci U S A. 2009;106(37):15967–15972. doi: 10.1073/pnas.0901477106 19717456 PMC2747227

[24] Kikuma T, Ohneda M, Arioka M, et al. Functional analysis of the ATG8 homologue Aoatg8 and role of autophagy in differentiation and germination in *aspergillus oryzae*. Eukaryot Cell. 2006;5(8):1328–1336. doi: 10.1128/EC.00024-06 16896216 PMC1539149

[25] Lv W, Wang C, Yang N, et al. Genome-wide functional analysis reveals that autophagy is necessary for growth, sporulation, deoxynivalenol production and virulence in *fusarium graminearum*. Sci Rep. 2017;7(1):11062. doi: 10.1038/s41598-017-11640-z 28894236 PMC5594004

[26] Voigt O, Poggeler S. Autophagy genes Smatg8 and Smatg4 are required for fruiting-body development, vegetative growth and ascospore germination in the filamentous ascomycete *sordaria macrospora*. Autophagy. 2013;9(1):33–49. doi: 10.4161/auto.22398 23064313 PMC3542216

[27] Yue JY, Wang YJ, Jiao JL, et al. Silencing of ATG2 and ATG7 promotes programmed cell death in wheat via inhibition of autophagy under salt stress. Ecotoxicol Environ Saf. 2021;225:112761. doi: 10.1016/j.ecoenv.2021.112761 34509161

[28] Zhao X, Feng W, Zhu X, et al. Conserved autophagy pathway contributes to stress tolerance and virulence and differentially controls autophagic flux upon nutrient starvation in *Cryptococcus neoformans*. Front Microbiol. 2019;10:2690. doi: 10.3389/fmicb.2019.02690 32038502 PMC6988817

[29] Yamauchi S, Mano S, Oikawa K, et al. Autophagy controls reactive oxygen species homeostasis in guard cells that is essential for stomatal opening. Proc Natl Acad Sci U S A. 2019;116(38):19187–19192. doi: 10.1073/pnas.1910886116 31484757 PMC6754613

[30] Yoshimoto K, Jikumaru Y, Kamiya Y, et al. Autophagy negatively regulates cell death by controlling NPR1-dependent salicylic acid signaling during senescence and the innate immune response in Arabidopsis. Plant Cell. 2009;21(9):2914–2927. doi: 10.1105/tpc.109.068635 19773385 PMC2768913

[31] Wang Y, Nishimura MT, Zhao T, et al. ATG2, an autophagy-related protein, negatively affects powdery mildew resistance and mildew-induced cell death in Arabidopsis. Plant J. 2011a;68(1):74–87. doi: 10.1111/j.1365-313X.2011.04669.x 21645148

[32] Wang Y, Wu Y, Tang D. The autophagy gene, *ATG18a*, plays a negative role in powdery mildew resistance and mildew-induced cell death in Arabidopsis. Plant Signal Behav. 2011b;6(9):1408–1410. doi: 10.4161/psb.6.9.16967 21847024 PMC3258078

[33] Hashimi SM, Wu NN, Ran J, et al. Silencing autophagy-related gene 2 (ATG2) results in accelerated senescence and enhanced immunity in soybean. Int J Mol Sci. 2021;22(21):11749. doi: 10.3390/ijms222111749 34769178 PMC8584260

[34] Chen L, Zhang X, Wang W, et al. Network and role analysis of autophagy in *Phytophthora sojae*. Sci Rep. 2017;7(1):1879. doi: 10.1038/s41598-017-01988-7 28500315 PMC5431975

[35] Luo Q, Wang FX, Zhong NQ, et al. The role of autophagy during development of the oomycete pathogen *phytophthora infestans*. J Genet Genomics. 2014;41(4):225–228. doi: 10.1016/j.jgg.2014.03.004 24780621

[36] Wang J, Zhou G, Huang W, et al. Autophagy-related gene *PlAtg6a* is involved in mycelial growth, asexual reproduction and tolerance to salt and oxidative stresses in *Peronophythora litchii*. Int J Mol Sci. 2022;23(3):1839. doi: 10.3390/ijms23031839 35163762 PMC8836449

[37] Qiu M, Li Y, Ye W, et al. A CRISPR/Cas9-mediated in situ complementation method for *phytophthora sojae* mutants. Mol Plant Pathol. 2021;22(3):373–381. doi: 10.1111/mpp.13028 33484494 PMC7865083

[38] Fang Y, Cui L, Gu B, et al. Efficient genome editing in the Oomycete *Phytophthora sojae* using CRISPR/Cas9. Curr Protoc Microbiol. 2017;44(1):21–21A 21 26. doi: 10.1002/cpmc.25 28166383

[39] Livak KJ, Schmittgen TD. Analysis of relative gene expression data using Real-Time Quantitative PCR and the 2^−ΔΔCT^ method. Methods. 2001;25(4):402–408. doi: 10.1006/meth.2001.1262 11846609

[40] Huang J, Xi P, Deng Y, et al. The mitogen-activated protein kinase PlMAPK2 is involved in zoosporogenesis and pathogenicity of *Peronophythora litchii*. Int J Mol Sci. 2021;22(7):3524. doi: 10.3390/ijms22073524 33805371 PMC8036616

[41] Lin L, Ye W, Wu J, et al. The MADS-box transcription factor PsMAD1 is involved in zoosporogenesis and pathogenesis of *Phytophthora sojae*. Front Microbiol. 2018;9:2259. doi: 10.3389/fmicb.2018.02259 30319576 PMC6165875

[42] Contento AL, Xiong Y, Bassham DC. Visualization of autophagy in Arabidopsis using the fluorescent dye monodansylcadaverine and a GFP-AtATG8e fusion protein. Plant J. 2005;42(4):598–608. doi: 10.1111/j.1365-313X.2005.02396.x 15860017

[43] Hardham AR. Cell biology of plant-oomycete interactions. Cell Microbiol. 2007;9(1):31–39. doi: 10.1111/j.1462-5822.2006.00833.x 17081190

[44] Sun J, Gao Z, Zhang X, et al. Transcriptome analysis of *Phytophthora litchii* reveals pathogenicity arsenals and confirms taxonomic status. PloS One. 2017;12(6):e0178245. doi: 10.1371/journal.pone.0178245 28570700 PMC5453482

[45] Blanco FA, Judelson HS. A bZIP transcription factor from *Phytophthora* interacts with a protein kinase and is required for zoospore motility and plant infection. Mol Microbiol. 2005;56(3):638–648. doi: 10.1111/j.1365-2958.2005.04575.x 15819621

[46] Judelson HS, Blanco FA. The spores of *Phytophthora*: weapons of the plant destroyer. Nat Rev Microbiol. 2005;3(1):47–58. doi: 10.1038/nrmicro1064 15608699

[47] Shintani T, Suzuki K, Kamada Y, et al. Apg2p functions in autophagosome formation on the perivacuolar structure. J Biol Chem. 2001;276(32):30452–30460. doi: 10.1074/jbc.M102346200 11382761

[48] Wang CW, Kim J, Huang WP, et al. Apg2 is a novel protein required for the cytoplasm to vacuole targeting, autophagy, and pexophagy pathways. J Biol Chem. 2001;276(32):30442–30451. doi: 10.1074/jbc.M102342200 11382760 PMC2737745

[49] Velikkakath AK, Nishimura T, Oita E, et al. Mammalian Atg2 proteins are essential for autophagosome formation and important for regulation of size and distribution of lipid droplets. MboC. 2012;23(5):896–909. doi: 10.1091/mbc.e11-09-0785 22219374 PMC3290647

[50] Abeliovich H, Klionsky DJ. Autophagy in yeast: mechanistic insights and physiological function. Microbiol Mol Biol Rev. 2001;65(3):463–479. doi: 10.1128/MMBR.65.3.463-479.2001 11528006 PMC99037

[51] Levine B, Klionsky DJ. Development by self-digestion: molecular mechanisms and biological functions of autophagy. Dev Cell. 2004;6(4):463–477. doi: 10.1016/S1534-5807(04)00099-1 15068787

[52] Meijer AJ, Codogno P. Regulation and role of autophagy in mammalian cells. Int J Biochem Cell Biol. 2004;36(12):2445–2462. doi: 10.1016/j.biocel.2004.02.002 15325584

[53] Kikuma T, Kitamoto K. Analysis of autophagy in *aspergillus oryzae* by disruption of Aoatg13, Aoatg4, and Aoatg15 genes. FEMS Microbiol Lett. 2011;316(1):61–69. doi: 10.1111/j.1574-6968.2010.02192.x 21204928

[54] Pollack JK, Harris SD, Marten MR. Autophagy in filamentous fungi. Fungal Genet Biol. 2009;46(1):1–8. doi: 10.1016/j.fgb.2008.10.010 19010432

[55] Veneault-Fourrey C, Barooah M, Egan M, et al. Autophagic fungal cell death is necessary for infection by the rice blast fungus. Science. 2006;312(5773):580–583. doi: 10.1126/science.1124550 16645096

[56] Liu XH, Liu TB, Lin FC. Monitoring autophagy in *Magnaporthe oryzae*. Methods Enzymol. 2008;451:271–294. 19185727 10.1016/S0076-6879(08)03219-9

[57] Shi H, Yang Z, Huang J, et al. An effector of ‘candidatus liberibacter asiaticus’ manipulates autophagy to promote bacterial infection. J Exp Bot erad176. 2023a;74(15):4670–4684. doi: 10.1093/jxb/erad176 37166404

[58] Shi J, Gong Y, Shi H, et al. ‘Candidatus liberibacter asiaticus’ secretory protein SDE3 inhibits host autophagy to promote huanglongbing disease in citrus. Autophagy. 2023b;1–17. doi: 10.1080/15548627.2023.2278414 PMC10392736 37249424

[59] Testi S, ML K, Allasia V, et al. An oomycete effector impairs autophagy in evolutionary distant organisms and favors host infection. Cold Spring Harbor Lab. 2019;697136. doi:10.1101/697136

[60] Tyler BM. *Phytophthora sojae*: root rot pathogen of soybean and model oomycete. Mol Plant Pathol. 2007;8(1):1–8. doi: 10.1111/j.1364-3703.2006.00373.x 20507474

