# Supplementary material for: Autophagy-related protein PlATG2 regulates the vegetative growth, sporangial cleavage, autophagosome formation, and pathogenicity of peronophythora litchii
Source: Virulence. 2024 Mar 4;15(1):2322183. doi: 10.1080/21505594.2024.2322183 (PMC10913709; doi:10.1080/21505594.2024.2322183)
Supplement: PlAtg2 supplementary materialclean.docx [file KVIR_A_2322183_SM0784.docx]

**Autophagy-related protein PlATG2 regulates the vegetative growth, sporangial cleavage, autophagosome formation, and pathogenicity of *Peronophythora litchii***

Lin Lv^a,b^**^#^**, Chengdong Yang^a,b^**^#^**, Xue Zhang^a,b^, Taixu Chen^a,b^, Manfei Luo^a,b^, Ge Yu^a,b^, Qinghe Chen^a,b^*

^a^Hainan Yazhou Bay Seed Laboratory, College of Breeding and Multiplication (Sanya Institute of Breeding and Multiplication), Hainan University, Sanya, China; ^b^Key Laboratory of Green Prevention and Control of Tropical Plant Diseases and Pests, Ministry of Education, School of Tropical Agriculture and Forestry, Hainan University, Haikou, China.

^#^These authors contributed equally to this work.

*Authors for Correspondence: Dr. Qinghe Chen

E-mail: qhchen@hainanu.edu.cn

**Running title: PlATG2 in *Peronophythora litchii***

**Key words: Autophagy; PlATG2; sporangial cleavage; autophagosome formation, pathogenicity, *Peronophythora litchii***

**
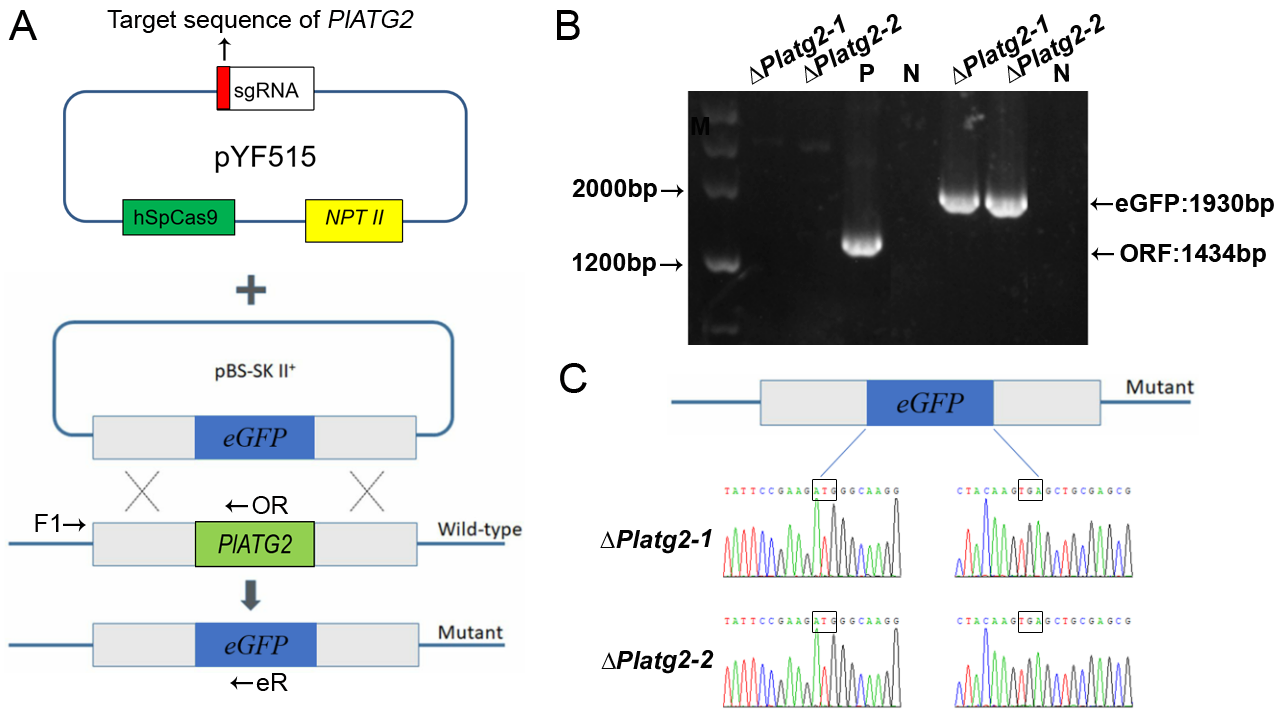
**

**Figure S1 Identification of CRISPR/Cas9-mediated** ***PlATG2* deletion mutants**

1. Schematic representation of the CRISPR/Cas9-mediated replacement strategy of *PlATG2* and plasmids used in transformation of *P. litchii*. (B) PCR identification of *PlATG2* deletion mutants with specific primer pairs indicated in (A). F1/OR and F1/eR primer pairs were used to identify the ORF of *PlATG2* was replaced by eGFP. Primer pairs listed in Table S1. (C) Sanger sequencing traces of junction regions demonstrated that the *PlATG2* ORF was completely replaced by the eGFP. Start and stop codons of eGFP are indicated using black box.

**Figure S2.** Expression levels of *PlATG2* in the *PlATG2* deletion mutants.

The transcription levels of *PlATG2* in SHS3, Δ*Platg2-1*, and Δ*Platg2-2* strains were determined by qRT-PCR with *β-ACTIN* as endogenous reference gene, validating *PlATG2* is barely expressed in the *PlATG2* deletion mutants. (** = p≤0.01).


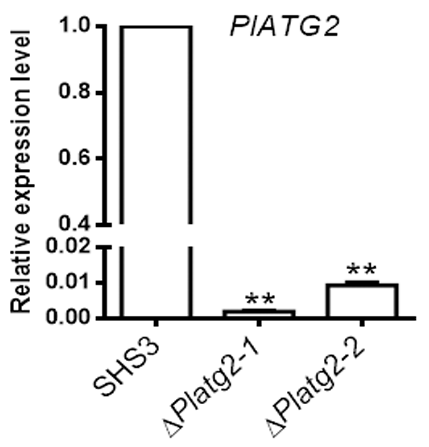


**Table S1** List of primer pairs used in this study

| Primers | Sequences（5’ → 3’） | Function |
| --- | --- | --- |
| *sgPlATG2-*1F | CTAGCGCTACGCTGATGAGTCCGTGAGGACGAAACGAGTAAGCTCGTCCGTAGCTGCCAAGACCATTG | *PlATG2-*sgRNA1-Cas9 expression plasmid |
| *sgPlATG2-*1R | AAACCAATGGTCTTGGCAGCTACGGACGAGCTTACTCGTTTCGTCCTCACGGACTCATCAGCGTAGCG |  |
| *sgPlATG2-*2F | CTAGCCGCGTCCTGATGAGTCCGTGAGGACGAAACGAGTAAGCTCGTCGACGCGGGTGGAGGTATTGG | *PlATG2-*sgRNA2-Cas9 expression plasmid |
| *sgPlATG2-*2R | AAACCCAATACCTCCACCCGCGTCGACGAGCTTACTCGTTTCGTCCTCACGGACTCATCAGGACGCGG |  |
| *pBS-PlATG2-*LF | CCCCTCGAGGTCGACGGTATGGCGTGTAAGGAAGTAAC | upstream sequence of *PlATG2* for gene replacement plasmid |
| *pBS-PlATG2-*LR | CCTTGCCCATCTTCGGAATAAAATATCAAATACTC |  |
| *pBS-eGFP-*F | TATTCCGAAGATGGGCAAGGGCGAGGAA | Donor DNA for gene replacement plasmid |
| *pBS-eGFP-*R | CGCTCGCAGCTCACTTGTAGAGTTCATCCATGCC |  |
| *pBS-PlATG2-*RF | CTACAAGTGAGCTGCGAGCGCGTACACA | downstream sequence of *PlATG2* for gene replacement plasmid |
| *pBS-PlATG2-*RR | CGGCCGCTCTAGAACTAGTGCGTGTTGCCTGAGGTGACAG |  |
| F1 | TACCAGGAACAGGAGAAGC | Identification of *PlATG2* mutants |
| OR | CACCTCGGCGTTCAGCAA |  |
| eR | GATGAACTCTACAAGTGA |  |
| *PlATG2 qrt F* | GAGTGAGCGAGAAGATGA | Expression level of *PlATG2* |
| *PlATG2 qrt R* | CGAGCAACGAGTAATGTG |  |
| *PlACTIN qrt F* | TCACGCTATTGTTCGTCTGG | Expression of *PlACTIN* |
| *PlACTIN qrt R* | TCATCTCCTGGTCAGAGTCC |  |
